# Contributions of phenotypic integration, plasticity and genetic adaptation to adaptive capacity relating to drought in *Banksia marginata* (Proteaceae)

**DOI:** 10.3389/fpls.2023.1150116

**Published:** 2023-04-21

**Authors:** Osazee O. Oyanoghafo, Adam D. Miller, Madeline Toomey, Collin W. Ahrens, David T. Tissue, Paul D. Rymer

**Affiliations:** ^1^ Hawkesbury Institute for the Environment, Western Sydney University, Richmond, NSW, Australia; ^2^ Department of Plant Biology and Biotechnology, Faculty of Life Sciences, University of Benin, Benin, Nigeria; ^3^ School of Life and Environmental Sciences, Deakin University, Princes Highway, Warrnambool, VIC, Australia; ^4^ Cesar Australia, Brunswick, VIC, Australia; ^5^ Global Centre for Land-Based Innovation, Western Sydney University, Richmond, NSW, Australia

**Keywords:** adaptive capacity, Banksia, functional traits, local adaptation, physiology, plasticity, Proteaceae

## Abstract

The frequency and intensity of drought events are predicted to increase because of climate change, threatening biodiversity and terrestrial ecosystems in many parts of the world. Drought has already led to declines in functionally important tree species, which are documented in dieback events, shifts in species distributions, local extinctions, and compromised ecosystem function. Understanding whether tree species possess the capacity to adapt to future drought conditions is a major conservation challenge. In this study, we assess the capacity of a functionally important plant species from south-eastern Australia (*Banksia marginata*, Proteaceae) to adapt to water-limited environments. A water-manipulated common garden experiment was used to test for phenotypic plasticity and genetic adaptation in seedlings sourced from seven provenances of contrasting climate-origins (wet and dry). We found evidence of local adaptation relating to plant growth investment strategies with populations from drier climate-origins showing greater growth in well-watered conditions. The results also revealed that environment drives variation in physiological (stomatal conductance, predawn and midday water potential) and structural traits (wood density, leaf dry matter content). Finally, these results indicate that traits are coordinated to optimize conservation of water under water-limited conditions and that trait coordination (phenotypic integration) does not constrain phenotypic plasticity. Overall, this study provides evidence for adaptive capacity relating to drought conditions in *B. marginata*, and a basis for predicting the response to climate change in this functionally important plant species.

## Introduction

Climate-induced drought events are projected to be more frequent and intense in the future, causing forest dieback and tree mortality events across the world, shifting species distributions and leading to extinctions (Engelbrecht et al., 2007; Goulden and Bales, 2019; Brodribb et al., 2020; Powers et al., 2020; Peters et al., 2021). Drought-related tree mortality has been recorded across continents and biomes with negative consequences on primary productivity and ecosystem functionality (Phillips et al., 2009; Allen et al., 2010; Nardini et al., 2013; Moore et al., 2016; Duke et al., 2017). A major conservation challenge is to understand the vulnerability of natural populations along with the capacity of tree species to adapt to increasing pressures associated with drought.

Plant tolerance to drought stress depends on different functional, structural and physiological traits. For instance, reduction in precipitation levels and associated decreased soil water potential, leads to negative plant water potential resulting in loss in cell turgor, which triggers the closure of stomata as a first response to water stress (Morgan, 1984; Tombesi et al., 2015). However, stomatal closure comes with associated costs, including reduction in photosynthesis and evaporative cooling as well as greater photoreceptive damage (Schulze, 1986; Pirasteh-Anosheh et al., 2016; Buckley, 2019; Henry et al., 2019). To compensate for the immediate impact of water limitation with stomatal closure, plants may adjust functional traits (morpho-physio-phenological traits) (Violle et al., 2007), prioritize resource allocation to different plant organs, and alter their allometry for efficient resource capture, conservation and protection (Eziz et al., 2017; Dai et al., 2020). For example, reduction in leaf size and shoot growth, increased sapwood to leaf area ratio (Huber value, HV), and root to shoot ratio can help to mitigate soil water shortages, while increasing wood density for mechanical support against xylem implosion from negative pressure (Gotsch et al., 2010; Keeley et al., 2011; Eziz et al., 2017; Liang et al., 2021). If water potential continues to decline, irrespective of the trait adjustment, gas bubbles (emboli) develop in the xylem vessel, leading to loss in xylem hydraulic collapse and eventually plant mortality under prolonged drought conditions (Choat et al., 2012; Choat et al., 2018). However, species may avoid this threshold if they can adapt to climate change through a combination of plastic and genetic mechanisms.

The adaptive capacity of a species is defined by its ability to adjust to climate or environmental change by shifting functional traits to enhance growth and survival (Williams et al., 2008; Foden et al., 2019). Trait changes can occur in direct response to environmental change (i.e. phenotypic plasticity) and also result from genetic differences between individual genotypes and populations (i.e. genetic adaptation; Nicotra et al., 2010). Populations from different climate-origins may be locally adapted to their environment and display differential trait expressions when exposed to contrasting environmental conditions. Characterising these differences is key to understanding historical evolutionary responses to environmental change and predicting responses to future environmental challenges (Nicotra and Davidson, 2010; Nicotra et al., 2010; Aspinwall et al., 2015). Experiments aimed at testing for variation in phenotypic plasticity and genetic adaptation among conspecific populations from different climate-origins are essential for determining the availability of genetic variation for adaptation to future climates (Ahrens et al., 2021).

Common garden experiments and reciprocal transplant studies are powerful tools applied in ecological research programs for characterising adaptive genetic differences among plant populations by testing for differential trait expression under controlled environmental conditions (genotype-by-environment interaction, G x E). Such studies involve growing single populations under varying conditions (to test for environmental effect, E), multiple populations with varying climate-origins under controlled common conditions (to test for genetic adaptation or effect, G) or contrasting conditions to test for interactions between genotype and environment, G x E (De Villemereuil et al., 2016). Local adaptation is inferred when higher fitness is observed in populations grown under conditions similar to its climate origin (Leimu and Fischer, 2008; Hereford, 2009). Several studies have documented differential trait expression among populations from different climate-origins; however, there is still a gap in knowledge surrounding the adaptive nature of trait plasticity (Lamy et al., 2014; Mclean et al., 2014; Drake et al., 2015; López et al., 2016; Blackman et al., 2017; Challis et al., 2022). There is mounting evidence relating to the importance of intra-specific genetic variation for adaptation to climate change, yet knowledge of the relative roles of phenotypic plasticity and genetic adaptation to drought is still lacking. Furthermore, the ability of plants to evolve drought tolerance may be constrained by trade-offs (Ramírez-Valiente and Cavender-Bares, 2017) and other interdependent physiological, structural, and growth traits.

Traits are coordinated as part of plant strategies to enhance growth and survival under their growth conditions; however, trait coordination (here after phenotypic integration) can constrain the ability for individual traits to respond to environmental variation through phenotypic plasticity (Gianoli and Palacio-López, 2009; Matesanz et al., 2010). Gianoli and Palacio-López (2009) previously reported that plasticity in response to drought treatment was constrained in key functional traits (e.g. specific leaf area, SLA) by the magnitude of associations with other traits (i.e. trait integration) in two perennial species (*Convolvulus chilensis*, *Lippia alba*). Indeed, plants adapted to dry conditions are known to have high trait coordination with conservative attributes (e.g. wood density) constraining growth plasticity (e.g. plant height) in response to high water availability (Kunstler et al., 2015; Nabais et al., 2018), while wet-origin plants are typically more plastic, allowing them to take advantage of additional resources (Münzbergová et al., 2017). As such, a negative correlation is typically expected between trait integration and plasticity (Gianoli and Palacio-López, 2009). However, Matesanz et al. (2021) uncovered a positive relationship between trait integration and phenotypic plasticity using drought response data of a Mediterranean shrub, *Lepidium subulatum*. Consequently, further investigation is needed to determine if this pattern exists in other plant systems, which traits may be coordinated with enhanced plasticity, and under what environment conditions integrated plastic traits might be favourable.

*Banksia* is a diverse genus in the Proteaceae with species found across the Australian continent including mesic wet forests, heathlands, and semi-arid open woodlands (George, 1999). *Banksia marginata* Cav., a functionally important tree species from south-eastern Australian, commonly known as the silver banksia, is a species occurring in savannas and forests. Populations are distributed widely and span a large climatic area including wet-temperate and warm-arid environments, making the species an ideal candidate for exploring phenotypic plasticity and genetic adaptation of drought-related traits. The objective of the study was to disentangle the effects of environment and genotype on trait expression, along with phenotypic plasticity and integration across seven *B. marginata* populations with contrasting climate-origins (wet and dry) grown under water-limited conditions in a common garden experiment.

Under common garden conditions, we measured growth traits (plant height, total leaf area, and basal diameter), structural and allocation traits (wood density, specific leaf area and leaf dry matter content), and physiological traits (stomatal conductance, predawn, midday water potential and relative chlorophyll content) to test for local adaptation in coordinated drought-related traits. We hypothesized that: i) there will be evidence of local adaptation to wet/dry climate-origins (G x E interaction) such that wet-origin populations grown under well-watered conditions will have greater growth compared to dry-origin populations; ii) structural allocation traits will be determined by climate-origin (G; genotype), such that wood density and leaf dry matter content will be greater in dry-origin populations; iii) physiological traits will be determined by the water treatment (E; environment), such that stomatal conductance and water potential will be reduced under water-limited conditions; iv) trait plasticity will be predicted by climate-origin, such that plasticity will be greatest in wet-origins; and v) trait-integration will constrain phenotypic plasticity, such that greater integration will reduce trait plasticity. This study provides novel empirical data regarding adaptive capacity to climate-induced drought, which is critical for predicting future adaptive responses in this functionally important plant species (Nicotra et al., 2010; Bongers et al., 2017).

## Materials and methods

### Seed collection and sowing

We selected seven naturally occurring populations of *B. marginata* with contrasting climate-origins (wet and dry) partitioned by precipitation of warmest quarter (PWQ) (**Table S1**, supplementary information). Seeds were collected from natural populations from distinct maternal individuals, dried and stored at room temperature. Seeds were sown on 29 January 2020, initially under nursery conditions in Hiko planting trays at the South West TAFE, Sherwood Park Campus growth facility (Warrnambool, Victoria, Australia). After establishment, seedlings were planted into 20 cm pots containing native potting mix (Bio Grow, Mt Gambier – Banksia/Grevillea mix) on 11^th^ of September 2020.

### Common garden experimental design

We conducted a water-controlled glasshouse experiment with seven populations of *B. marginata* using a split block design. The glasshouse regulated temperature through roof vents, misting, and a hydronic pump system (mean temperature 18.9 °C and relative humidity of 57%). Pots were randomly arranged in blocks on pallets (1.1 m x 1.1 m) with two replicates of each population, except FUR which only had a single plant on each pallet due to low germination success. The design consisted of 16 pallets in the growth facility with 4 rows with 4 pallets per row, which was surrounded by a border row of *B. marginata* plants (not included in data collection) to minimize potential edge effects on the study plants. Pallets were assigned to well-watered and water reduction (water-limited) treatments, alternating treatments between neighbouring pallets. This resulted in 16 replicates per population per treatment (i.e. a total of at least 32 individuals per population), except one population (FUR) with 8 replicates per treatment (16 individuals). Some plants were lost from the experiment as they did not establish or were impacted from other factors (e.g. insect) as such at completion, BAY, JIL, LMS, and MHA populations had 20 individuals while COL, FUR, and WLT had 10, 12, and 15 individuals respectively (i.e. 117 established plants).

Initially, plants were watered by hand twice a week to allow for sapling acclimatisation during the establishment phase. Drippers delivering 3 L/h on 4-way manifolds, connected to 5 mm tubing running along each row from a programmable controller were established. All plants were well watered for 6 weeks before the water treatment was imposed. The frequency and duration of watering events (irrigation) supplied to each pot was adjusted to achieve the significant differences in plant water availability realised in differential growth responses. Plants assigned to the well-watered treatment received irrigation five times per week for 15 minutes (*ca.* 935 ml per week), while those assigned to the water-limited treatment were irrigated three times per week for seven minutes (*ca.* 261 ml per week). The well-watered and water-limited treatments received an average water supply of 7.02% (weighing 90 g) and 3.43% (weighing 47 g) respectively. This design was implemented to mimic prolonged water limited stress but to avoid plant death.

### Trait measurements

Plant trait measurements were conducted after 129-151 days in the treatment period. Plant growth, functional traits, and *in-situ* measurements were conducted as follows.

#### Plant growth

Plant size was measured non-destructively on 14th May 2021 (151 days) on all established plants as indicator of growth. Plant height (H_max_, mm) was measured with a ruler starting from the soil level to the highest tip of the plant. Basal diameter (BD, mm) at the base of each plant was obtained using a digital calliper as an average of two perpendicular measures. Total leaf area (TLA, cm ^2^) was estimated as the total leaf count multiplied by the average leaf size obtained from the leaf area meter (see functional traits below).

#### Plant functional traits

Sub-samples of plants were used to estimate functional traits on 14 May 2021 (151 days). Leaf area was estimated on a sub-sample of 10 leaves (fully expanded sun-lit) using a leaf area meter (Li-Cor 3100, LI-COR, Lincoln, NE, USA). Leaf samples were weighed to determine fresh mass and then leaf samples were oven-dried at 70°C for 48 h to obtain dry mass; these data were used to determine leaf dry matter content (LDMC = dry mass (g)/fresh mass (g)). Specific leaf area (SLA) was measured using five (5) well-developed and healthy leaves from five (5) replicate plants per population per treatment were sampled during destructive harvest, scanned through the leaf area meter, and dry mass obtained using the oven. Specific leaf area (SLA) was estimated as the leaf area over the dry mass of the leaf sample [leaf area (cm^2^)/dry mass (g)]. Wood density was obtained through destructive harvest of the 117 established plants Wood density [stem dry mass (g)/stem volume (cm^3^)] was estimated on a 5 cm standard length from the base of the main stem. Wood volume obtained using the formula: V= (0.5D) ^2^ × π × L (Pérez-Harguindeguy et al., 2013), where D is the stem diameter with bark removed measured by averaging three digital calliper measurements (top, middle and bottom), and L is the stem length. Wood samples were subsequently oven-dried for 105°C for 72 h and weighed to determine the stem dry mass.

#### In-situ measures

Water potential, Ψ (predawn, PD and midday MD, -MPa), stomatal conductance (gs), and relative chlorophyll content (RC) were measured on 22 April 2021 (129 days). A single fully expanded, sun-lit, leaf from five (5) replicates per population per treatment was sampled at predawn (*ca* 1 hour before sunrise) and midday to obtain leaf water potentials using a Scholander pressure chamber. Stomatal conductance was measured on an adjacent leaf on the same individuals between 10 am and 1:00 pm using a leaf porometer (SC-1 Leaf Porometer) at relative humidity 50-80%. Relative chlorophyll content (RC) was measured using a Digital PhotosynQ device (MultispeQ V.2.0), which is a modified version of the Soil Plant Analysis Development (SPAD) chlorophyll meter (Markwell et al., 1995; Kuhlgert et al., 2016).

### Phenotypic plasticity and phenotypic integration

We considered plasticity as trait variation among well-watered and water-limited treatments for each population. Plasticity was calculated by the formula: PP= |(xMax-xMin)/xMax| , where PP is plasticity index, x is trait, while xMax and xMin are maximum and minimum mean trait values for each population per treatment (Valladares et al., 2000; Valladares et al., 2006; Granata et al., 2020). The index ranges from 0-1, where plasticity index closer to 1 indicates the trait is more plastic.

Phenotypic integration (PI) was estimated as the number of significant correlations a trait has with all other traits, as discussed by Matesanz et al. (2021). Separate estimates of PI were generated for populations from wet and dry climate-origins, as well as PI for the combined dataset. Phenotypic integration (PI) was determined based on trait pair-wise Pearson’s correlation where the number of significant relationships (P< 0.05) was summed for each trait.

### Statistical analysis

Linear mixed effect models were used to investigate the significance of genetic or climate-origin (G) and environmental or water treatment factor (E), along with the genotype-by-environment interaction (G x E), while controlling for spatial variation in the glasshouse and populations sampled within climate-origins. The independent fixed factors were climate-origin (G) (wet, dry) and water treatment (E) (WW, WL), while planting block (pallet) and population were used as random variables in the mixed effects model conducted using the lmer function in the *R* package (Bates et al., 2015). Model residuals were inspected; appropriate data transformations and removal of extreme outliers were performed where necessary. Kenward Roger degrees of freedom approximation was used to obtain the analysis of variance (ANOVA) for the mixed effects models. We used *post-hoc* Tukey tests to determine significant differences between climate-origins and treatments using the ‘emmeans’ R package (Lenth et al., 2020). Principal component analysis (PCA) was used to determine levels of trait coordination between selected traits and climatic variables obtained from Worldclim using *rda* function in *vegan R* statistics package. Probability level of 95% was used to draw the ellipses in the PCA. Trait correlations was further tested using through bivariate linear models.

## Results

### Growth traits

**Figure 1:**
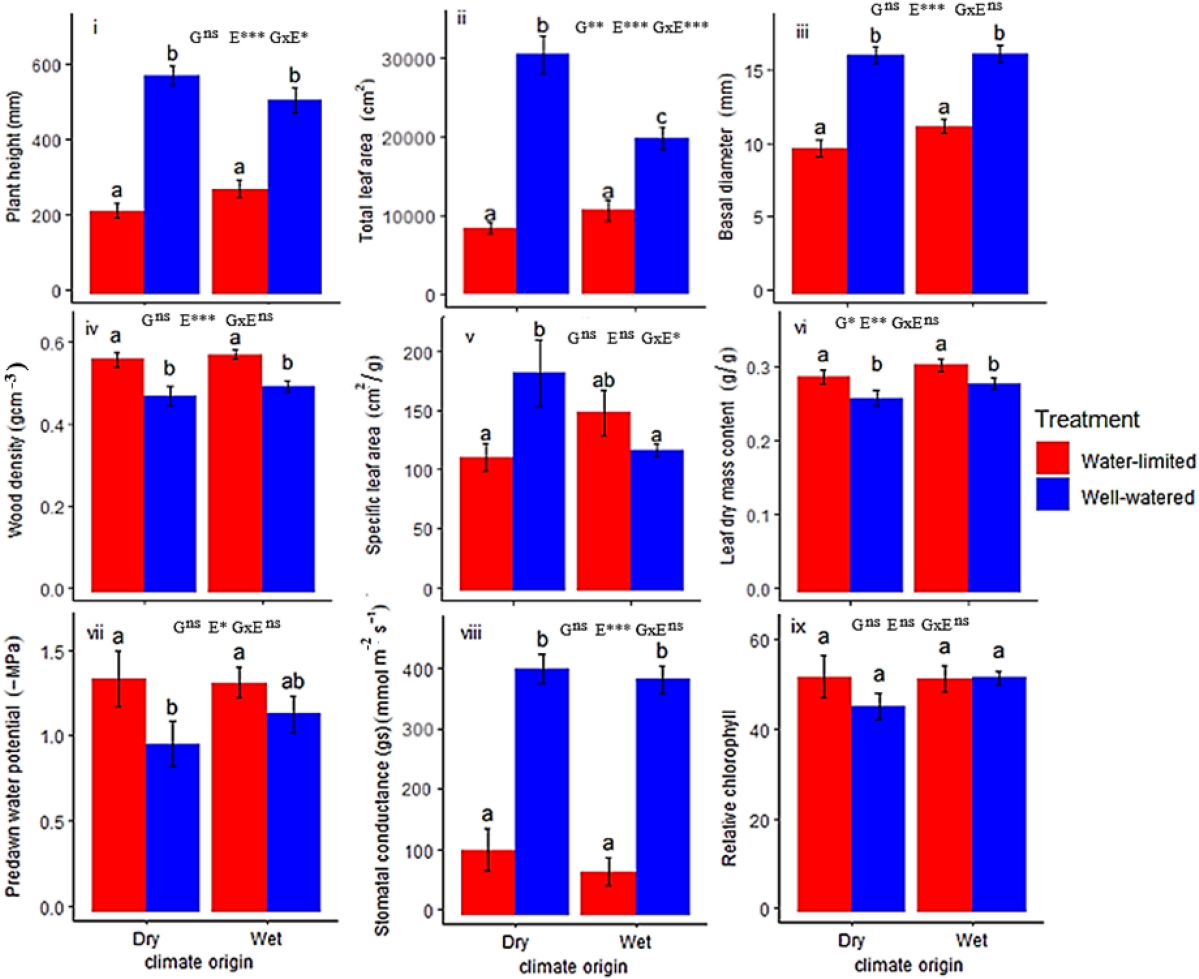
Trait expressions of wet and dry climate-origin populations under water-limited (WL) and well-watered (WW) treatments. G means genotype differences (or climate-origin differences), while E means environment (treatment differences). G x E means interaction between G and E i.e., genotype-by-environment. Significant codes: 0 ‘***’ 0.001 ‘**’ 0.01 ‘*’ 0.05 ns not-sigificant. The results of the Tukey posthoc test are shown with letters above the bars. Different letters are significantly different, while the same letter is statistically similar.

Plant height (H_max_) was determined by environment (E) and genotype-by-environment interaction (G x E). Total leaf area (TLA) was determined by genetic differences (G), environment (E) and G x E, while basal diameter was determined by E only (**Figure 1**; **Table 1**). H_max_, TLA and BD were significantly higher under well-watered (WW) conditions (560.32 ± 25.08 mm, 24975.49 ± 1688.52 cm^2^, 16.58 ± 0.47 mm, respectively) compared to water-limited (WL) conditions (257.48 ± 17.52 mm, 9655.47 ± 834.55 cm^2^ and 10.89 ± 0.37 mm, respectively). Dry-origin populations had greater but not significant H_max_ (597.96 ± 30.68 mm), and TLA (21843.92 ± 2808.32 cm^2^) under WW conditions compared to wet-origin populations (H_max_, 533.43 ± 36.68 mm; TLA, 16426 ± 1403.43 cm^2^, **Table 1**). Conversely, dry-origin populations had lower (but not significant) H_max_ (221.5 ± 19.38 mm) and TLA (8288.82 ± 708.38 cm^2^) in WL conditions compared to wet-origin populations (H_max_, 280.76 ± 25.41 mm and TLA, 10601.61 ± 1284.01 mm^2^, **Table 1**). However, TLA was higher in dry vs wet populations only in WW conditions (**Figure 1**).

**Table 1:** Analysis of variance testing for effect of water treatment (environment, E), climate-origin (genotype, G) and treatment and climate origin interaction (G x E) on trait expression.

Response (Abbreviation)	Statistic	Treatment	Climate-Origin	Treatment x Climate-Origin
Plant height	F	100.119	0.031	4.566
(Hmax)	P	0.000***	0.861	0.035*
Total leaf area	F	53.922	9.359	14.249
(TLA)	P	0.000***	0.003**	0.000***
Basal diameter	F	101.135	1.936	1.478
(BD)	P	0.000***	0.167	0.227
Wood density	F	33.097	1.153	0.259
(WD)	P	0.000***	0.286	0.612
Specific leaf area	F	1.111	1.104	5.860
(SLA)	P	0.324	0.298	0.019*
Leaf dry matter content	F	8.903	4.089	0.059
(LDMC)	P	0.018*	0.048*	0.809
Stomatal conductance	F	84.837	1.030	0.066
(gs)	P	0.000***	0.317	0.798
Predawn water potential	F	5.363	0.239	0.654
(PD)	P	0.028*	0.646	0.425
Midday water potential	F	7.634	0.880	0.199
(MD)	P	0.009**	0.392	0.658
Relative Chlorophyll	F	1.939	3.240	0.547
(RC)	P	0.212	0.082	0.466

Significant codes: 0 ‘***’ 0.001 ‘**’ 0.01 ‘*’ 0.05. Degree of freedom is 1 for all factors.

### Structural and physiological traits

Variation in structural traits (WD and LDMC) were determined by water treatment differences (E) except in leaf dry matter (LDMC) where climate-origin (G) effect was also significant (**Figure 1**; **Table 1**). Differences in wood density was driven by E, such that we observed significantly higher WD (0.61 ± 0.03 g cm^-3^) in WL conditions compared to WW conditions (0.51 ± 0.02 g cm^-3^, **Figure 1**). Differences in LDMC were determined by both G and E (**Figure 1**; **Table 1**). We observed significant higher LDMC (0.30 ± 0.01 g/g) in WL conditions compared to WW conditions (0.27 ± 0.01 g/g), and LDMC was significantly higher in wet-origin (0.294 ± 0.01 g/g) than dry-origin populations (0.274 ± 0.01 g/g). We observed significant G x E interaction in SLA (**Figure 1**; **Table 1**). In WW conditions, SLA was significantly greater in dry-origin populations (184.79 ± 28.19 cm^2^/g) compared to wet-origin populations (119.28 ± 5.28), but in WL conditions, dry-origin populations (113.29 ± 11.99) had lower (though not significant) SLA than wet-origin populations (151.29 ± 19.35).

Variation in physiological traits (*g*_s_, PD and MD) were determined by treatment differences (E), except relative chlorophyll (RC) which had limited variation irrespective of the environment and genotype (**Figure 1**; **Table 1**; MD **Figure S1**). *g*_s_ was significantly higher in WW conditions (399.9 ± 15.8 mmol m^-2^ s^-1^) compared to WL conditions (121.1 ± 28.2 mmol m^-2^ s^-1^), indicative of stomatal regulation conserving water under water limitation. While water potentials (PD and MD) were significantly lower (greater negative potential) in WL conditions (PD, -1.36 ± 0.08 MPa; MD, -1.89 ± 0.07 MPa) compared to WW conditions (PD, -1.06 ± 0.08 MPa; MD, -1.60 ± 0.06 MPa) (**Figure S1**), these values indicate the plants adjusted to the drought conditions, limiting water loss and growth. No significant climate-origin (G) or interaction (G x E) effects were detected for physiological traits.

### Trait coordination and correlation

**Figure 2:**
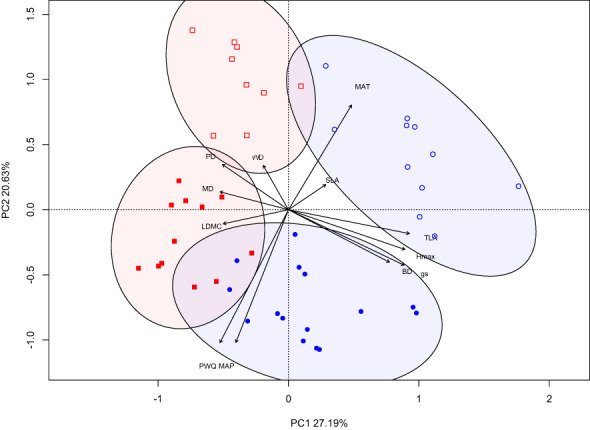
PCA analysis of plant traits for wet and dry origin populations grown under different water treatments. Open and closed shape signifies dry and wet-climate-origin, while blue-circle and red-square signifies water-limited and well-watered treatments.

The principal components analysis (PCA) revealed distinct clusters for each climate-origin and treatment combination based on the two main axes of variation (PC1 and PC2), accounting for 47.8% of the total variation (**Figure 2**). PC1 accounted for 27.2% of total variation, and was associated positively with H_max_, BD, TLA, *g*_s_ and negatively with PD, MD and LDMC. In contrast, PC2 accounted for 20.6% of the total variation, was positively associated with WD, PD, MAT and negatively with *g*_s_, MAP and PWQ (**Figure 2**). The PCA shows separation of climate-origin (wet/dry) on the y-axis (PC2) and separation of the treatments on x-axis (PC1). The dry-origin has relatively well defined (separate) treatment groups compared to wet-origin with overlap WW and WL treatments groups (**Figure 2**).

**Figure 3:**
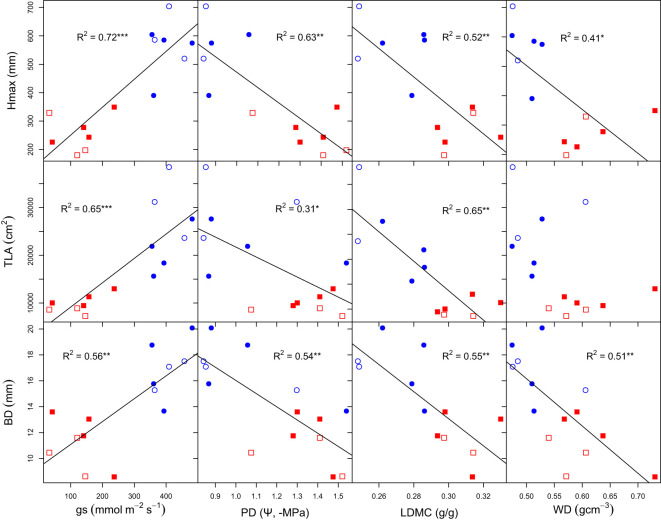
Bivariate relationships of growth, morphological and physiological traits. Hmax, maximum plant height; gs, stomatal conductance; WD, wood density; PD, predawn water potential; TLA, total leaf area; TLA, total leaf area; BD, basal diameter and LDMC, leaf dry matter content. Open and closed shape signifies dry and wet climate-origin, while blue-circle and red-square signifies water-limited and well-watered treatments. R2 adjusted R-squared. Significant codes: 0 ‘***’ 0.001 ‘**’ 0.01 ‘*’ 0.05.

Trait relationships were explicitly explored using bivariate linear models. We found that H_max_ was positively related to gs (R^2^ = 72, P = 0.000) and negatively related to PD, LDMC and WD (R^2^ = 63 P = 0.001, R^2^ = 52 P = 0.007, R^2^ = 41 P = 0.019, **Figure 3**). TLA was also positively related to *g*_s_ (R^2^ = 65 P = 0.000) and negatively related to PD and LDMC (R^2^ = 31 P = 0.03, R^2^ = 65 P = 0.001), but not related to WD (R^2^ = 15 P = 0.09). Similarly, BD was positively related to *g*_s_ (R^2^ = 56 P = 0.001) and negatively related to PD, LDMC and WD (R^2^ = 54 P = 0.002, R^2^ = 55 P = 0.004, R^2^ = 51 P = 0.003).

### Phenotypic plasticity and its relationship with phenotypic integrations

**Figure 4:**
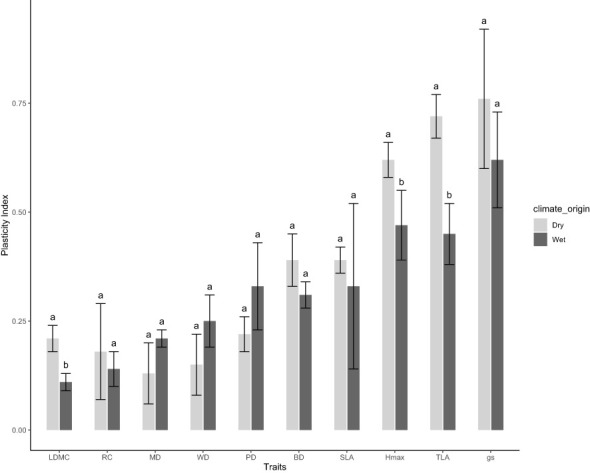
Variation in phenotypic plasticity between wet and dry climate-origin. H_max_ = maximum plant height, gs = stomatal conductance, WD, wood density; PD, predawn water potential; MD, midday water potential; TLA, total leaf area; SLA, specific leaf area; BD, basal diameter and LDMC, leaf dry matter content. Letter signifies differences in trait plasticity, while dark and grey colours represents wet and dry climate-origin, respectively.

We observed trait plasticity in response to water treatments, however, the level of plasticity varied among traits and climate-origins (**Figure 4**). Across climate-origin, gs had the highest plasticity index (0.69) followed by TLA (0.57) and H_max_ (0.55), while RC and LDMC had the least plasticity (0.16) followed by MD (0.17) and WD (0.20) (**Figure 4**). There was a significant difference among climate-origins in the level of plasticity found for growth traits (H_max_ and TLA) and the structural allocation trait, LDMC, such that dry-origin had significant higher plasticity in these traits than wet-origin (**Figure 4**). While not significant this trend for greater plasticity in dry-origin plants was observed for all other traits, except leaf water potentials (MD, PD) and WD where plants from wet-origins tended to have more plasticity.

**Figure 5:**
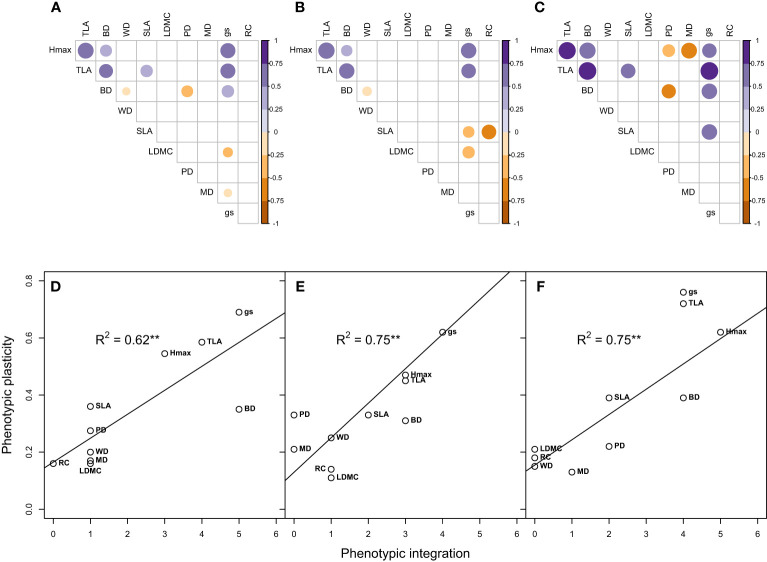
Trait integration and its relationship with phenotypic plasticity across wet and dry climate-origin **(A, D)**, within wet-origin **(B, E)** and dry-origin **(C, F)**. Each point represents a trait. Hmax, maximum plant height; gs, stomatal conductance; WD, wood density; PD, predawn water potential; MD, midday water potential; TLA, total leaf area; SLA, specific leaf area; BD, basal diameter and LDMC, leaf dry matter content. R2 adjusted R-squared. Significant codes: 0 ‘***’ 0.001 ‘**’ 0.01 ‘*’ 0.05.

In terms of overall trait integration, *g*_s_ and BD had the highest trait associations (5), followed by TLA (4), while RC had the least (zero) with no association with any other traits (**Figure 5A**). These patterns of trait integration were largely maintained for wet and dry climate-origins (**Figures 5B, C**) with most traits having similar levels of integration ( ± 1) except for H_max_ and PD which both showed increased (+2) trait integration in dry-origin plants (**Figures 5A–C**). We found that all growth-related traits had a significant positive relationship with each other (H_max_ vs TLA, R^2^ = 76, P= 0.001; H_max_ vs BD, R^2^ = 45, P= 0.04; TLA vs BD, R^2^ = 53, P= 0.02).

We observed significant associations between trait plasticity and integration even in populations varying in climate-origin, such that trait plasticity was predicted by trait integration (wet and dry origin R^2^ = 0.62; wet-origin, R^2^ = 0.75, dry-origin R^2^ = 0.75; **Figures 5D–F** respectively). In all comparisons, *g*_s_ had high plasticity and integration, moderate-high for growth traits (H_max_, TLA, BD), and RC, MP, WD and LDMC had low plasticity and integration.

## Discussion

The objective of the study was to investigate the influence of phenotypic integration, plasticity and genetic adaptation on adaptive capacity relating to drought in *B. marginata*. To achieve this, we explored the effects of environment and genotype on trait expression, as well as trait integration, across wet- and dry-origin populations of *B. marginata* grown under water-manipulated common garden conditions. We provide evidence of differential responses to water limitation among populations from contrasting climate-origins, indicating significant genotype x environment (G x E) interactions and genetically determined adaptive differences among *B. marginata* populations. We also demonstrate significant trait plasticity, such that drier origin populations had greater plasticity in growth (H_max_ and TLA) and structural (LDMC) traits than wetter origin populations. Our findings also suggest that traits were coordinated to optimize water conservation under water-limited conditions, and that phenotypic integration does not limit plasticity of traits. Overall, this study provides evidence for adaptive capacity to drought in *B. marginata*, and a basis for predicting future adaptive responses to climate change in this functionally important plant species.

### Evidence of local adaptation in growth investment strategies

Local adaptation to different environments is a significant process leading to ecological specialization in plants (Vanwallendael et al., 2019). Trade-offs in resource allocation to enhance growth or persistence have been well recognised in locally adapted populations with different exposure to drought (MacTavish and Anderson, 2020). Our common garden experiment revealed differential responses to water limitation among *B. marginata* populations varying in climate-origins and provides evidence for local adaptation relating to drought. We found significant G x E interactions in growth (H_max_, TLA) and allocation (SLA) (**Table 1**), suggesting a possible differential responses in carbon investment. Specifically, dry population had higher TLA and SLA in WW conditions compared to wet population suggesting that when water is not limiting, dry populations invest lower carbon for leaf construction and can produce more leaves or leaves with a higher area. While there was no significant differences in growth, the reduction in H_max_, TLA, and SLA under WL conditions in dry populations compared to wet populations could suggest that genotypes from drier climate-origin may have reduced investment in growth under WL. These findings are supported by other studies on woody plants showing G x E patterns across species climatic distributions (Ahrens et al., 2020; Challis et al., 2022). This demonstrates evidence of local adaptation in growth strategies under water-limited conditions among *B. marginata* populations and suggests a trade-off between leaf area for growth and investment in structural tissue for resilience under water-limited conditions in drier populations. While we did not find support for G x E patterns in structural (WD and LDMC) and physiological (stomatal conductance*, g*_s_ and water potential) traits, other studies have previously found them to be associated with local adaptation (Malan and Verryn, 1996; Lima et al., 2000; El-Soda et al., 2014; Challis et al., 2022). These findings also indicate that plants from dry climate-origin could have potentially evolved genotypes that allow them to cope with water stress by employing conservative growth strategies, but also to switch to resource utilization (i.e. greater growth) under favourable conditions. However, this may also enhance the vulnerability of drought-adapted populations in cases where abundant rainfall during the growth phase is followed by drought conditions.

### Determinant of plant growth

Plant growth has previously been suggested to be linked to environmental variability, with reduced growth often characteristic of resource limitation (i.e. arid environments; Chaves et al., 2002). Similar to previous studies, we found growth traits (H_max_, TLA and BD) were determined by environment (**Figure 1**; **Table 1**), such that reduced growth was observed under water limited conditions compared to well-watered conditions (Moles et al., 2009; Wright et al., 2017; Henn et al., 2018; Zhu et al., 2020). This highlights the potential for shifts in climatic variables (e.g. rainfall) to influence the performance of *B. marginata* populations spanning the species’ distribution.

Our findings support theoretical and empirical studies (Drake et al., 2013; Li et al., 2018; Buckley, 2019; Duursma et al., 2019) in showing that *g*_s_ is correlated with plant size, such that growth rates decline with greater stomatal regulation under water-limited conditions. The reduction in *g*_s_ under water stress conditions results in reduced carbon uptake critical for growth and helps to reduce exposure to further water loss through evapo-transpiration while increasing allocation for structural traits (LDMC and WD, **Figure 3**). Indeed, we also found support for structural traits determining growth, such that in water-limited conditions, plants tend to have conservative attributes (e.g. WD and LDMC) constraining growth. This suggests there is a trade-off between growth and structural traits, which is driven by environmental conditions (Roderick and Berry, 2001; Fajardo, 2022). However, there is need for further research to more fully understand other mechanisms influencing plant growth under water-limited conditions (e.g. rooting structures and allocation of biomass above and below ground).

### Determinant of drought tolerance traits (physiological and structural)

We found physiological (*g*_s_, *Ψ* PD) and structural traits (WD, LDMC) were directly influenced by environment (E), suggesting the potential for trait shifts with climate (e.g. rainfall). The pattern observed in our study clearly conforms to the broader scientific literature, showing that water limitation results in decline in *Ψ*, reduction in *g*_s_ and increase in WD (Hacke et al., 2001; Roderick and Berry, 2001; McCulloh et al., 2011; Liang et al., 2021). For example, under water stress conditions, water potential (Ψ a good indicator of physiological stress) gradually declines, increasing the negative pressure of the xylem leading to a potential loss of hydraulic conductivity (Choat et al., 2018; Liang et al., 2021). To prevent the continuous decline of xylem water potential, plants tend to adjust traits that can maintain internal water balance. Reduction in *g*_s_ is often the first line of response to prevent further water loss and the decline of xylem water potential (Tombesi et al., 2015; Buckley, 2019). While our treatment was not designed to cause critical water stress (indicated by the MD Ψ > 2 MPa), we were successful in reducing growth and observed a large reduction in *g*_s_, along with increased allocation to structural tissues, in water-limited plants. The reduction in *g*_s_ under water limitation has significant implications on carbon assimilation and allocation for structural reinforcement.

Structural traits account for carbon investment in construction of water conducting tissues enhancing tolerance to water stress at expense of growth in stress conditions (Chave et al., 2009; Martínez-Cabrera et al., 2009; Liang et al., 2021). Under water stress conditions plants tend to allocate more resources for construction of denser structural tissues (e.g. denser wood and leaf tissue) as smaller plants with greater structural allocation would be less vulnerable to droughts (Hacke et al., 2001; Pittermann et al., 2006; Sperry et al., 2006; Lauder et al., 2019; Fajardo, 2022). WD is regarded to be an important structural trait indicating drought resistance (Hacke et al., 2001; Greenwood et al., 2017; Rosner, 2017; Liang et al., 2021). Contrary to our expectation that WD would be genetically determined (Lenz et al., 2010; Soro et al., 2022), we found that WD was determined by environment (E), such that water-limited plants had greater WD compared to well-watered plants. This demonstrates that wood density may be plastic in response to shifts in water supply. Similar findings have also been reported with water limitation acting as determinant of WD, with denser wood often portrayed as mechanical and structural reinforcement to prevent xylem implosion resulting from decline in water potential (Hacke et al., 2001; Searson et al., 2004; Onoda et al., 2010; Markesteijn et al., 2011). In support of the trade-off mechanism between growth and structural investment, we also found that LDMC was determined by environment (E), such that water-limited plants had greater LDMC compared to well-watered plants (**Figure 1**). This further demonstrates that structural traits are plastic in response to sustained periods of water shortage. Hence, plants may be able to shift plastically from growth to structural investment to persist in dry regions.

Structural traits are thought to be genetically determined and influenced by selection to optimise growth and structural support under varying rainfall conditions. While WD was not found to differ between wet and dry climate-origins, it has been found to be a heritable trait in woody plants (Lenz et al., 2010; Ahrens et al., 2020; Soro et al., 2022). In addition to environment determining structural traits, we found the leaf construction cost trait (LDMC) (Grassein et al., 2010) was also determined by climate-origin (G). This suggests that variation in LDMC may be partly heritable and genetically controlled. Wet-origin plants had greater LDMC (i.e. less water content) compared to dry climate-origins, suggesting a slow investment-return strategy in wetter origins (Zhu et al., 2020).

### Traits are coordinated mechanistically as a whole-plant strategy

Trait expressions are important in defining species ecological strategies. Thus, understanding the constraints on trait variation under different environments may provide useful insights into how species respond to climate change. In this study, we provide evidence that intraspecific trait expressions are coordinated and partitioned with the primary axis of trait variation associated with water-treatment and the secondary axis associated with climate-origin (see multivariate PCA analysis). This demonstrates that trait expressions are closely aligned to form functional axes of specializations either for resource conservation or utilization (Maire et al., 2013; Díaz et al., 2016).

The influence of environment in shaping the coordination of water-dependent and tolerance traits (e.g. MD, PD, LDMC and WD) defines the avoidance strategies a species may employ under water limited conditions. Our common garden experiment showed *g*_s_ to be negatively associated with tolerance traits, indicating lesser priority for carbon uptake for growth compared to hydraulic safety (Scholz et al., 2008). Plants exposed to water-limited conditions tended to invest more in the development of denser stems and leaves, while regulating growth and exposure to evapotranspiration *via* reduced carbon uptake (i.e. decreased *g*_s_). Conversely, under well-watered conditions, growth traits (TLA, H_max_, and BD) and *g*_s_ were coordinated as a rapid water utilisation strategy, such that plants with greater *g*_s_ had greater investment in growth. In support of our findings, larger plants tend to have increased *g*_s_ associated with increased photosynthesis and respiration critical for the development of growth tissues (Wong et al., 1979; Henry et al., 2019). Furthermore, results from the bivariate relationships confirm there was a mechanistic switch in strategy from rapid growth to slow growth under water limited conditions. Adjusting stomatal conductance mechanistically was critical to controlling resource investment for growth or tolerance, as *g*_s_ was positively related to all growth traits and negatively to tolerance traits. Hence, reduced *g*_s_ was associated with declines in water potential and greater LDMC. Higher LDMC highlights the absence of intercellular space and high mesophyll tissue resistance to gas diffusion, thus reducing leaf transpiration (Liu et al., 2019).

A signature of climate-origin is evident in the multivariate PCA analysis with defined clusters for plants from wet- and dry-origins. While the differences among climate-origins is predominantly driven by climatic predictors, the second principal component is also positively associated with WD and PD. Interestingly, the water treatment differences are more distinct for plants from dry-origins compared to wet-origin plants which show partial overlap in trait space. This indicates the possibility of greater plasticity in growth and structural traits in dry-origin compared to wet-origin plants (Kreyling et al., 2019). The differences in plasticity may be attributed to climatic differences driving local genetic adaptation among populations (Kingsolver and Buckley, 2017). Our findings highlight that trait coordination influences plant responses to water limitation, revealing the importance of incorporating varied traits and genotypes when accounting for different strategies in predicting species responses to climate change.

### Variation in trait plasticity between wet and dry climate-origin

Theory predicts that climatic variability selects for genotypes that facilitate greater plasticity (Matesanz et al., 2010; Dostál et al., 2016; Carvajal et al., 2017; Vázquez et al., 2017; Stotz et al., 2021). Drier populations usually experience greater variability in rainfall and temperature, occupying the lowest and upper continuum in species climatic ranges, respectively. This appears to apply to *B. marginata*, as we found that populations from dry climate-origin had greater plasticity in growth (H_max_ and TLA) and structural allocation (LDMC) traits compared to those of wet-origins. Overall, this suggests that drier populations may possess the capacity to adapt to future climate change through phenotypic plasticity (Alvarez-Maldini et al., 2020). Across climate-origin, *g*_s_ was more plastic while LDMC was least plastic, suggesting higher level of *g*_s_ variability compared to structural traits (e.g. WD, LDMC) in response to shift in water availability. In support of our findings, studies have shown that phenotypic plasticity in physiological traits (*g*_s_) are greater than structural leaf and wood traits (Bongers et al., 2017). This is true as physiological traits (e.g. *g*_s_) rely on regulatory mechanism, often flexible and easily adjustable by plants in response to environmental stimulus to avoid stress but structural traits are more fixed with limited opportunity for dynamic change, often related to ontogeny and long-term growth conditions (Quero et al., 2006; Bongers et al., 2017). This variation in trait plasticity may constrain the expression of trait variation where other dependent traits are less plastic and limit plants ability to respond to drought.

### Phenotypic integration (PI) does not constrain phenotypic plasticity (PP)

We found evidence that phenotypic integration (i.e. correlation with other traits) does not limit the ability to express plastic responses. Unlike previous studies that have indicated trait integration to constrains plasticity in some plant species (Gianoli and Palacio-López, 2009; Matesanz et al., 2010). We observed a positive relationship between PI and PP suggesting plasticity increases with trait integration across different climate-origins. A similar finding was reported in a recent study by Matesanz et al. (2021) showing that trait plasticity was positively related to trait integration. This suggests that PI-PP association may be complementary and provide an alternative strategy for plants to adapt to different climate-origins. Traits that are strongly integrated were more plastic than less integrated traits. This implies that suites of highly plastic and integrated traits shift together in a coordinated way to variation in water availability in contrast to poorly integrated traits. Our findings have implications in reshaping the old-theory that PP and PI are alternative mechanisms to incorporate the co-adaptation of interdependent traits in response to environmental change. However, care should be taken in scaling this finding to the global scale as genetic differences in some traits could influence the pattern observed.

## Conclusions

This study highlights the importance of environment (E), genotype (G) and their interaction (G x E) in shaping trait expression in *B. marginata*, which is crucial for predicting the response of this functionally important tree species under climate change. We found evidence of local adaptation associated with growth (H_max_, TLA and SLA), signifying the potential of adaptive strategies for shifting investment from growth to structural tissues through stomatal regulation as a buffer to changes in water availability, particularly in drier-origin populations. Water availability had a significant influence on the expression of physiological, structural, growth and allocation traits (*g*_s_, WD, H_max_, TLA, BD and LDMC), demonstrating the potential of changing climates to impact species performance and distribution patterns. We found traits to be coordinated mechanistically as a whole-plant response to water-availability, and that dry-origin populations are more plastic than those of wet-origin, suggesting that drier populations are locally adapted and less vulnerable to drought conditions. This study also provides evidence to reject the theory of plasticity being constrained by trait-integration, suggesting some plant traits work in coordination to respond to shifts in water availability. Further studies to test the generality of this phenomenon across populations and species is needed, along with exploration of broader trait pairs, including above and belowground traits. Overall, our data highlights to adaptive capacity of species to persist under climate change through plasticity of coordinated traits shifting in concert as a mechanism for species survival to future drought events.

## Data availability statement

The raw data supporting the conclusions of this article will be made available by the authors, without undue reservation.

## Author contributions

OO and PR designed and conceptualized the research idea, OO, AM, MT, CA and PR conducted the experiment and collected data, OO analyzed and presented data, OO, AM, M.T, C.A, DT, and P.R interpreted data and findings, OO prepared the manuscript while OO, AM, MT, CA, DT, and PR reviewed, edited and contributed critical comments to drafts and approved the manuscript. All authors contributed to the article and approved the submitted version.

## Supplementary material

Supplementary Table 1 Natural occurring populations of *B. marginata*, with their locations and climate-origin selected for the study. LAT, latitude; LONG, longitude; MAT, mean annual temperature; MAP, mean annual precipitation; PWQ, precipitation of the warmest quarter.

Supplementary Figure 1 Mid-day water potential (MD) expressions of wet and dry climate-origin populations under water-limited (WL) and well-watered (WL) treatments.

## References

[B1] Ahrens C. W. Andrew M. E. Mazanec R. A. Ruthrof K. X. Challis A. Hardy G.. (2020). Plant functional traits differ in adaptability and are predicted to be differentially affected by climate change. Ecol. Evol. 10, 232–248. doi: 10.1002/ece3.5890 31988725 PMC6972804

[B2] Ahrens C. W. Rymer P. D. Tissue D. T. (2021). Intra-specific trait variation remains hidden in the environment. New Phytol. 229, 1183–1185. doi: 10.1111/nph.16959 33105042

[B3] Allen C. D. Macalady A. K. Chenchouni H. Bachelet D. McDowell N. Vennetier M.. (2010). A global overview of drought and heat-induced tree mortality reveals emerging climate change risks for forests. For. Ecol. Manage. 259, 660–684. doi: 10.1016/j.foreco.2009.09.001

[B4] Alvarez-Maldini C. Acevedo M. Dumroese R. K. González M. Cartes E. (2020). Intraspecific variation in drought response of three populations of cryptocarya alba and persea lingue, two native species from Mediterranean central Chile. Front. Plant Sci. 11. doi: 10.3389/fpls.2020.01042 PMC7378861 32765551

[B5] Aspinwall M. J. Loik M. E. Resco de Dios V. Tjoelker M. G. Payton P. R. Tissue D. T. (2015). Utilizing intraspecific variation in phenotypic plasticity to bolster agricultural and forest productivity under climate change. Plant Cell Environ. 38, 1752–1764. doi: 10.1111/pce.12424 25132508

[B6] Bates D. Mächler M. Bolker B. Walker S. (2015). Fitting linear mixed-effects models using lme4. J. Stat. Softw. 67 (1), 1–48. doi: 10.18637/jss.v067.i01

[B7] Blackman C. J. Aspinwall M. J. Tissue D. T. Rymer P. D. (2017). Genetic adaptation and phenotypic plasticity contribute to greater leaf hydraulic tolerance in response to drought in warmer climates. Tree Physiol. 37, 583–592. doi: 10.1093/treephys/tpx005 28338733

[B8] Bongers F. J. Olmo M. Lopez-Iglesias B. Anten N. P. R. Villar R. (2017). Drought responses, phenotypic plasticity and survival of Mediterranean species in two different microclimatic sites. Plant Biol. 19, 386–395. doi: 10.1111/PLB.12544 28054449

[B9] Brodribb T. J. Powers J. Cochard H. Choat B. (2020). Hanging by a thread? forests and drought. Science 368, 261–266. doi: 10.1126/science.aat7631 32299945

[B10] Buckley T. N. (2019). How do stomata respond to water status? New Phytol. 224, 21–36. doi: 10.1111/nph.15899 31069803

[B11] Carvajal D. E. Loayza A. P. Rios R. S. Gianoli E. Squeo F. A. (2017). Population variation in drought-resistance strategies in a desert shrub along an aridity gradient: Interplay between phenotypic plasticity and ecotypic differentiation. Perspect. Plant Ecology Evol. Systematics 29, 12–19. doi: 10.1016/j.ppees.2017.10.001

[B12] Challis A. Blackman C. Ahrens C. Medlyn B. Rymer P. Tissue D. (2022). Adaptive plasticity in plant traits increases time to hydraulic failure under drought in a foundation tree. Tree Physiol. 42, 708–721. doi: 10.1093/treephys/tpab096 34312674

[B13] Chave J. Coomes D. Jansen S. Lewis S. L. Swenson N. G. Zanne A. E. (2009). Towards a worldwide wood economics spectrum. Ecol. Lett. 12, 351–366. doi: 10.1111/j.1461-0248.2009.01285.x 19243406

[B14] Chaves M. M. Pereira J. S. Maroco J. Rodrigues M. L. Ricardo C. P. P. Osório M. L.. (2002). How plants cope with water stress in the field. photosynthesis and growth. Ann. Bot. 89, 907–916. doi: 10.1093/aob/mcf105 12102516 PMC4233809

[B15] Choat B. Brodribb T. J. Brodersen C. R. Duursma R. A. López R. Medlyn B. E. (2018). Triggers of tree mortality under drought. Nature 558, 531–539. doi: 10.1038/s41586-018-0240-x 29950621

[B16] Choat B. Jansen S. Brodribb T. J. Cochard H. Delzon S. Bhaskar R.. (2012). Global convergence in the vulnerability of forests to drought. Nature 491, 752–755. doi: 10.1038/nature11688 23172141

[B17] Dai L. Guo X. Ke X. Lan Y. Zhang F. Li Y.. (2020). Biomass allocation and productivity–richness relationship across four grassland types at the qinghai plateau. Ecol. Evol. 10, 506–516. doi: 10.1002/ece3.5920 31988738 PMC6972799

[B18] De Villemereuil P. Gaggiotti O. E. Mouterde M. Till-Bottraud I. (2016). Common garden experiments in the genomic era: New perspectives and opportunities. Heredity 116, 249–254. doi: 10.1038/hdy.2015.93 26486610 PMC4806574

[B19] Díaz S. Kattge J. Cornelissen J. H. C. Wright I. J. Lavorel S. Dray S.. (2016). The global spectrum of plant form and function. Nature 529, 167–171. doi: 10.1038/nature16489 26700811

[B20] Dostál P. Fischer M. Prati D. (2016). Phenotypic plasticity is a negative, though weak, predictor of the commonness of 105 grassland species. Global Ecol. Biogeography 25, 464–474. doi: 10.1111/geb.12429

[B21] Drake J. E. Aspinwall M. J. Pfautsch S. Rymer P. D. Reich P. B. Smith R. A.. (2015). The capacity to cope with climate warming declines from temperate to tropical latitudes in two widely distributed eucalyptus species. Global Change Biol. 21, 459–472. doi: 10.1111/gcb.12729 25378195

[B22] Drake P. L. Froend R. H. Franks P. J. (2013). Smaller, faster stomata: Scaling of stomatal size, rate of response, and stomatal conductance. J. Exp. Bot. 64, 495–505. doi: 10.1093/jxb/ers347 23264516 PMC3542046

[B23] Duke N. C. Kovacs J. M. Griffiths A. D. Preece L. Hill D. J. E. Van Oosterzee P.. (2017). Large-Scale dieback of mangroves in australia’s gulf of carpentaria: A severe ecosystem response, coincidental with an unusually extreme weather event. Mar. Freshw. Res. 68, 1816–1829. doi: 10.1071/MF16322

[B24] Duursma R. A. Blackman C. J. Lopéz R. Martin-StPaul N. K. Cochard H. Medlyn B. E. (2019). On the minimum leaf conductance: its role in models of plant water use, and ecological and environmental controls. New Phytol. 221, 693–705. doi: 10.1111/nph.15395 30144393

[B25] El-Soda M. Boer M. P. Bagheri H. Hanhart C. J. Koornneef M. Aarts M. G. M. (2014). Genotype-environment interactions affecting preflowering physiological and morphological traits of brassica rapa grown in two watering regimes. J. Exp. Bot. 65, 697–708. doi: 10.1093/jxb/ert434 24474811 PMC3904722

[B26] Engelbrecht B. M. J. Comita L. S. Condit R. Kursar T. A. Tyree M. T. Turner B. L.. (2007). Drought sensitivity shapes species distribution patterns in tropical forests. Nature 447, 80–82. doi: 10.1038/nature05747 17476266

[B27] Eziz A. Yan Z. Tian D. Han W. Tang Z. Fang J. (2017). Drought effect on plant biomass allocation: A meta-analysis. Ecol. Evol. 7, 11002–11010. doi: 10.1002/ece3.3630 29299276 PMC5743700

[B28] Fajardo A. (2022). Wood density relates negatively to maximum plant height across major angiosperm and gymnosperm orders. Am. J. Bot. 109, 250–258. doi: 10.1002/ajb2.1805 34766624

[B29] Foden W. B. Young B. E. Akçakaya H. R. Garcia R. A. Hoffmann A. A. Stein B. A.. (2019). Climate change vulnerability assessment of species. Wiley Interdiscip. Reviews: Climate Change 10, e551. doi: 10.1002/wcc.551

[B30] George A. S. (1999). “Banksia,” in In flora of Australia: Volume 17B: Proteaceae 3: Hakea to dryandra. Ed. Wilson A. (Melbourne: CSIRO Publishing / Australian Biological Resources Study), 175–251.

[B31] Gianoli E. Palacio-López K. (2009). Phenotypic integration may constrain phenotypic plasticity in plants. Oikos 118, 1924–1928. doi: 10.1111/j.1600-0706.2009.17884.x

[B32] Gotsch S. G. Geiger E. L. Franco A. C. Goldstein G. Meinzer F. C. Hoffmann W. A. (2010). Allocation to leaf area and sapwood area affects water relations of co-occurring savanna and forest trees. Oecologia 163, 291–301. doi: 10.1007/s00442-009-1543-2 20058025

[B33] Goulden M. L. Bales R. C. (2019). California Forest die-off linked to multi-year deep soil drying in 2012–2015 drought. Nat. Geosci. 12, 632–637. doi: 10.1038/s41561-019-0388-5

[B34] Granata M. U. Bracco F. Catoni R. (2020). Phenotypic plasticity of two invasive alien plant species inside a deciduous forest in a strict nature reserve in Italy. J. Sustain. Forestry 39, 346–364. doi: 10.1080/10549811.2019.1670678

[B35] Grassein F. Till-Bottraud I. Lavorel S. (2010). Plant resource-use strategies: The importance of phenotypic plasticity in response to a productivity gradient for two subalpine species. Ann. Bot. 106, 637–645. doi: 10.1093/aob/mcq154 20682576 PMC2944977

[B36] Greenwood S. Ruiz-Benito P. Martínez-Vilalta J. Lloret F. Kitzberger T. Allen C. D.. (2017). Tree mortality across biomes is promoted by drought intensity, lower wood density and higher specific leaf area. Ecol. Lett. 20, 539–553. doi: 10.1111/ele.12748 28220612

[B37] Hacke U. G. Sperry J. S. Pockman W. T. Davis S. D. McCulloh K. A. (2001). Trends in wood density and structure are linked to prevention of xylem implosion by negative pressure. Oecologia 126, 457–461. doi: 10.1007/s004420100628 28547229

[B38] Henn J. J. Buzzard V. Enquist B. J. Halbritter A. H. Klanderud K. Maitner B. S.. (2018) Intraspecific trait variation and phenotypic plasticity mediate alpine plant species response to climate change Front. Plant Sci. 871 1548 doi: 10.3389/fpls.2018.01548 PMC6243391 30483276

[B39] Henry C. John G. P. Pan R. Bartlett M. K. Fletcher L. R. Scoffoni C.. (2019). A stomatal safety-efficiency trade-off constrains responses to leaf dehydration. Nat. Commun. 10, 1–9. doi: 10.1038/s41467-019-11006-1 31363097 PMC6667445

[B40] Hereford J. (2009). A quantitative survey of local adaptation and fitness trade-offs. Am. Nat. 173, 579–588. doi: 10.1086/597611 19272016

[B41] Keeley J. E. Bond W. J. Bradstock R. A. Pausas J. G. Rundel P. W. (2011). Fire in mediterranean ecosystems: Ecology, evolution and management. Fire Mediterr. Ecosystems: Ecology Evol. Manage. 9780521824, 1–515. doi: 10.1017/CBO9781139033091

[B42] Kingsolver J. G. Buckley L. B. (2017). Evolution of plasticity and adaptive responses to climate change along climate gradients. Proc. R. Soc. B. 284, 20170386. doi: 10.1098/rspb.2017.0386 PMC5563792 28814652

[B43] Kreyling J. Puechmaille S. J. Malyshev A. V. Valladares F. (2019). Phenotypic plasticity closely linked to climate at origin and resulting in increased mortality under warming and frost stress in a common grass. Ecol. Evol. 9, 1344–1352. doi: 10.1002/ece3.4848 30805164 PMC6374657

[B44] Kuhlgert S. Austic G. Zegarac R. Osei-Bonsu I. Hoh D. Chilvers M. I.. (2016). MultispeQ beta: A tool for large-scale plant phenotyping connected to the open photosynQ network. R. Soc. Open Sci. 3, 160592. doi: 10.1098/rsos.160592 27853580 PMC5099005

[B45] Kunstler G. Falster D. Coomes D. A. Hui F. Kooyman R. M. Laughlin D. C.. (2015). Plant functional traits have globally consistent effects on competition. Nat. 2015 529:7585 529, 204–207. doi: 10.1038/nature16476 26700807

[B46] Lamy J. B. Delzon S. Bouche P. S. Alia R. Vendramin G. G. Cochard H.. (2014). Limited genetic variability and phenotypic plasticity detected for cavitation resistance in a Mediterranean pine. New Phytol. 201, 874–886. doi: 10.1111/nph.12556 24180459

[B47] Lauder J. D. Moran E. V. Hart S. C. (2019). Fight or flight? potential tradeoffs between drought defense and reproduction in conifers. Tree Physiol. 39, 1071–1085. doi: 10.1093/treephys/tpz031 30924877

[B48] Leimu R. Fischer M. (2008). A meta-analysis of local adaptation in plants. PloS One 3, e4010. doi: 10.1371/journal.pone.0004010 19104660 PMC2602971

[B49] Lenz P. Cloutier A. MacKay J. Beaulieu J. (2010). Genetic control of wood properties in picea glauca - an analysis of trends with cambial age. Can. J. For. Res. 40, 703–715. doi: 10.1139/X10-014

[B50] Lenth R. V. Bolker B. Buerkner P. Herve M. Herve M. Herve M.. (2020). Estimated Marginal Means, aka Least-Squares Means. R package 'version 1.8.5'. Available at: https://cran.r-project.org/web//packages/emmeans/emmeans.pdf.

[B51] Li X. Blackman C. J. Choat B. Duursma R. A. Rymer P. D. Medlyn B. E.. (2018). Tree hydraulic traits are coordinated and strongly linked to climate-of-origin across a rainfall gradient. Plant Cell Environ. 41, 646–660. doi: 10.1111/pce.13129 29314083

[B52] Liang X. Ye Q. Liu H. Brodribb T. J. (2021). Wood density predicts mortality threshold for diverse trees. New Phytol. 229, 3053–3057. doi: 10.1111/nph.17117 33251581

[B53] Lima J. T. Breese M. C. Cahalan C. M. (2000). Genotype-environment interaction in wood basic density of eucalyptus clones. Wood Sci. Technol. 34, 197–206. doi: 10.1007/s002260000041

[B54] Liu C. Li Y. Xu L. Chen Z. He N. (2019). Variation in leaf morphological, stomatal, and anatomical traits and their relationships in temperate and subtropical forests. Sci. Rep. 9, 1–8. doi: 10.1038/s41598-019-42335-2 30967600 PMC6456615

[B55] López R. Cano F. J. Choat B. Cochard H. Gil L. (2016). Plasticity in vulnerability to cavitation of pinus canariensis occurs only at the driest end of an aridity gradient. Front. Plant Sci. 7. doi: 10.3389/fpls.2016.00769 PMC4891331 27375637

[B56] MacTavish R. Anderson J. T. (2020). Resource availability alters fitness trade-offs: implications for evolution in stressful environments. Am. J. Bot. 107 (2), 308–318. doi: 10.1002/ajb2.1417 31943133

[B57] Maire V. Gross N. Hill D. Martin R. Wirth C. Wright I. J.. (2013). Disentangling coordination among functional traits using an individual-centred model: Impact on plant performance at intra- and inter-specific levels. PloS One 8 (10), e77372. doi: 10.1371/journal.pone.0077372 24130879 PMC3793938

[B58] Malan F. S. Verryn S. D. (1996). Effect of genotype-by-environment interaction on the wood properties and qualities of four-year-old eucalyptus grandis and e. grandis hybrids. South Afr. Forestry J. 176 (1), 47–53. doi: 10.1080/00382167.1996.9629709

[B59] Markesteijn L. Poorter L. Paz H. Sack L. Bongers F. (2011). Ecological differentiation in xylem cavitation resistance is associated with stem and leaf structural traits. Plant Cell Environ. 34, 137–148. doi: 10.1111/j.1365-3040.2010.02231.x 20946587

[B60] Markwell J. Osterman J. C. Mitchell J. L. (1995). Calibration of the minolta SPAD-502 leaf chlorophyll meter. Photosynthesis Res. 46, 467–472. doi: 10.1007/BF00032301 24301641

[B61] Martínez-Cabrera H. I. Jones C. S. Espino S. Jochen Schenk H. (2009). Wood anatomy and wood density in shrubs: Responses to varying aridity along transcontinental transects. Am. J. Bot. 96, 1388–1398. doi: 10.3732/ajb.0800237 21628286

[B62] Matesanz S. Blanco-Sánchez M. Ramos-Muñoz M. de la Cruz M. Benavides R. Escudero A. (2021). Phenotypic integration does not constrain phenotypic plasticity: differential plasticity of traits is associated to their integration across environments. New Phytol. 231, 2359–2370. doi: 10.1111/nph.17536 34097309

[B63] Matesanz S. Gianoli E. Valladares F. (2010). Global change and the evolution of phenotypic plasticity in plants. Ann. New York Acad. Sci. 1206, 35–55. doi: 10.1111/j.1749-6632.2010.05704.x 20860682

[B64] McCulloh K. A. Meinzer F. C. Sperry J. S. Lachenbruch B. Voelker S. L. Woodruff D. R.. (2011). Comparative hydraulic architecture of tropical tree species representing a range of successional stages and wood density. Oecologia 167, 27–37. doi: 10.1007/s00442-011-1973-5 21445684

[B65] Mclean E. H. Prober S. M. Stock W. D. Steane D. A. Potts B. M. Vaillancourt R. E.. (2014). Plasticity of functional traits varies clinally along a rainfall gradient in eucalyptus tricarpa. Plant Cell Environ. 37, 1440–1451. doi: 10.1111/pce.12251 24329726

[B66] Moles A. T. Warton D. I. Warman L. Swenson N. G. Laffan S. W. Zanne A. E.. (2009). Global patterns in plant height. J. Ecol. 97, 923–932. doi: 10.1111/j.1365-2745.2009.01526.x

[B67] Moore G. W. Edgar C. B. Vogel J. G. Washington-Allen R. A. March R. G. Zehnder R. (2016). Tree mortality from an exceptional drought spanning mesic to semiarid ecoregions. Ecol. Appl. 26, 602–611. doi: 10.1890/15-0330 27209798

[B68] Morgan J. M. (1984). Osmoregulation and water stress in higher plants. Annu. Rev. Plant Physiol. 35, 299–319. doi: 10.1146/annurev.pp.35.060184.001503

[B69] Münzbergová Z. Hadincová V. Skálová H. Vandvik V. (2017). Genetic differentiation and plasticity interact along temperature and precipitation gradients to determine plant performance under climate change. J. Ecol. 105, 1358–1373. doi: 10.1111/1365-2745.12762

[B70] Nabais C. Hansen J. K. David-Schwartz R. Klisz M. López R. Rozenberg P. (2018). The effect of climate on wood density: What provenance trials tell us? For. Ecol. Manage. 408, 148–156. doi: 10.1016/j.foreco.2017.10.040

[B71] Nardini A. Battistuzzo M. Savi T. (2013). Shoot desiccation and hydraulic failure in temperate woody angiosperms during an extreme summer drought. New Phytol. 200, 322–329. doi: 10.1111/nph.12288 23593942

[B72] Nicotra A. B. Atkin O. K. Bonser S. P. Davidson A. M. Finnegan E. J. Mathesius U.. (2010). Plant phenotypic plasticity in a changing climate. Trends Plant Sci. 15, 684–692. doi: 10.1016/j.tplants.2010.09.008 20970368

[B73] Nicotra A. B. Davidson A. (2010). Adaptive phenotypic plasticity and plant water use. Funct. Plant Biol. 37, 117–127. doi: 10.1071/FP09139

[B74] Onoda Y. Richards A. E. Westoby M. (2010). The relationship between stem biomechanics and wood density is modified by rainfall in 32 Australian woody plant species. New Phytol. 185, 493–501. doi: 10.1111/j.1469-8137.2009.03088.x 19925557

[B75] Pérez-Harguindeguy N. Díaz S. Garnier E. Lavorel S. Poorter H. Jaureguiberry P.. (2013). New handbook for standardised measurement of plant functional traits worldwide. Aust. J. Bot. 61, 167–234. doi: 10.1071/BT12225

[B76] Peters J. M. R. López R. Nolf M. Hutley L. B. Wardlaw T. Cernusak L. A.. (2021). Living on the edge: A continental-scale assessment of forest vulnerability to drought. Global Change Biol. 27, 3620–3641. doi: 10.1111/gcb.15641 33852767

[B77] Phillips O. L. Aragão L. E. O. C. Lewis S. L. Fisher J. B. Lloyd J. López-González G.. (2009). Drought sensitivity of the amazon rainforest. Science 323, 1344–1347. doi: 10.1126/science.1164033 19265020

[B78] Pirasteh-Anosheh H. Saed-Moucheshi A. Pakniyat H. Pessarakli M. (2016). Stomatal responses to drought stress. In Ahmad P. Ed. Water Stress and Crop Plants. doi: 10.1002/9781119054450.ch3

[B79] Pittermann J. Sperry J. S. Wheeler J. K. Hacke U. G. Sikkema E. H. (2006). Mechanical reinforcement of tracheids compromises the hydraulic efficiency of conifer xylem. Plant Cell Environ. 29, 1618–1628. doi: 10.1111/j.1365-3040.2006.01539.x 16898022

[B80] Powers J. S. Vargas G. G. Brodribb T. J. Schwartz N. B. Pérez-Aviles D. Smith-Martin C. M.. (2020). A catastrophic tropical drought kills hydraulically vulnerable tree species. Global Change Biol. 26, 3122–3133. doi: 10.1111/gcb.15037 32053250

[B81] Quero J. L. Villar R. Marañón T. Zamora R. (2006). Interactions of drought and shade effects on seedlings of four quercus species: Physiological and structural leaf responses. New Phytol. 170, 819–834. doi: 10.1111/j.1469-8137.2006.01713.x 16684241

[B82] Ramírez-Valiente J. A. Cavender-Bares J. (2017). Evolutionary trade-offs between drought resistance mechanisms across a precipitation gradient in a seasonally dry tropical oak (Quercus oleoides). Tree Physiol. 37, 889–901. doi: 10.1093/TREEPHYS/TPX040 28419347

[B83] Roderick M. L. Berry S. L. (2001). Linking wood density with tree growth and environment: A theoretical analysis based on the motion of water. New Phytol. 149, 473–485. doi: 10.1046/j.1469-8137.2001.00054.x 33873331

[B84] Rosner S. (2017). Wood density as a proxy for vulnerability to cavitation: Size matters. J. Plant Hydraulics 4, e001. doi: 10.20870/jph.2017.e001

[B85] Scholz F. G. Bucci S. J. Goldstein G. Meinzer F. C. Franco A. C. Salazar A. (2008). Plant- and stand-level variation in biophysical and physiological traits along tree density gradients in the cerrado. Braz. J. Plant Physiol. 20, 217–232. doi: 10.1590/s1677-04202008000300006

[B86] Schulze E. D. (1986). Carbon dioxide and water vapor exchange in response to drought in the atmosphere and in the soil. Annu. Rev. Plant Physiol. 37, 247–274. doi: 10.1146/annurev.pp.37.060186.001335

[B87] Searson M. J. Thomas D. S. Montagu K. D. Conroy J. P. (2004). Wood, density and anatomy of water-limited eucalypts. Tree Physiol. 24, 1295–1302. doi: 10.1093/treephys/24.11.1295 15339739

[B88] Soro A. Lenz P. Hassegawa M. Roussel J. R. Bousquet J. Achim A. (2022). Genetic influence on components of wood density variation in white spruce. Forestry 95, 153–165. doi: 10.1093/forestry/cpab044

[B89] Sperry J. S. Hacke U. G. Pittermann J. (2006). Size and function in conifer tracheids and angiosperm vessels. Am. J. Bot. 93, 1490–1500. doi: 10.3732/ajb.93.10.1490 21642096

[B90] Stotz G. C. Salgado-Luarte C. Escobedo V. M. Valladares F. Gianoli E. (2021). Global trends in phenotypic plasticity of plants. Ecol. Lett. 24, 2267–2281. doi: 10.1111/ele.13827 34216183

[B91] Tombesi S. Nardini A. Frioni T. Soccolini M. Zadra C. Farinelli D.. (2015). Stomatal closure is induced by hydraulic signals and maintained by ABA in drought-stressed grapevine. Sci. Rep. 5, 1–12. doi: 10.1038/srep12449 PMC4513549 26207993

[B92] Valladares F. Sanchez-Gomez D. Zavala M. A. (2006). Quantitative estimation of phenotypic plasticity: Bridging the gap between the evolutionary concept and its ecological applications. J. Ecol. 94, 1103–1116. doi: 10.1111/j.1365-2745.2006.01176.x

[B93] Valladares F. Wright S. J. Lasso E. Kitajima K. Pearcy R. W. (2000). Plastic phenotypic response to light of 16 congeneric shrubs from a panamanian rainforest. Ecology 81, 1925–1936. doi: 10.1890/0012-9658(2000)081[1925:PPRTLO]2.0.CO;2

[B94] Vanwallendael A. Soltani A. Emery N. C. Peixoto M. M. Olsen J. Lowry D. B. (2019). A molecular view of plant local adaptation: Incorporating stress-response networks. Annu. Rev. Plant Biol. 70, 559–583. doi: 10.1146/annurev-arplant-050718-100114 30786237

[B95] Vázquez D. P. Gianoli E. Morris W. F. Bozinovic F. (2017). Ecological and evolutionary impacts of changing climatic variability. Biol. Rev. 92, 22–42. doi: 10.1111/brv.12216 26290132

[B96] Violle C. Navas M. L. Vile D. Kazakou E. Fortunel C. Hummel I.. (2007). Let the concept of trait be functional! Oikos 116, 882–892. doi: 10.1111/j.0030-1299.2007.15559.x

[B97] Williams S. E. Shoo L. P. Isaac J. L. Hoffmann A. A. Langham G. (2008). Towards an integrated framework for assessing the vulnerability of species to climate change. PloS Biol. 6, e325. doi: 10.1371/journal.pbio.0060325 19108608 PMC2605927

[B98] Wong S. C. Cowan I. R. Farquhar G. D. (1979). Stomatal conductance correlates with photosynthetic capacity. Nature 282, 424–426. doi: 10.1038/282424a0

[B99] Wright I. J. Dong N. Maire V. Prentice I. C. Westoby M. Díaz S.. (2017). Global climatic drivers of leaf size. Science 357, 917–921. doi: 10.1126/science.aal4760 28860384

[B100] Zhu J. Zhu H. Cao Y. Li J. Zhu Q. Yao J.. (2020). Effect of simulated warming on leaf functional traits of urban greening plants. BMC Plant Biol. 20, 1–13. doi: 10.1186/s12870-020-02359-7 32245420 PMC7119294

