# Supplementary material for: Contributions of phenotypic integration, plasticity and genetic adaptation to adaptive capacity relating to drought in Banksia marginata (Proteaceae)
Source: Front Plant Sci. 2023 Apr 21;14:1150116. doi: 10.3389/fpls.2023.1150116 (PMC10160485; doi:10.3389/fpls.2023.1150116)
Supplement: Supplementary Table 1 — Natural occurring populations of B. marginata, with their locations and climate-origin selected for the study. LAT, latitude; LONG, longitude; MAT, mean annual temperature; MAP, mean annual precipitation; PWQ, precipitation of the warmest quarter. [file Table_1.docx]

TABLE 1 Analysis of variance testing for effect of water treatment (environment, E), climate-origin (genotype, G) and treatment and climate origin interaction (G x E) on trait expression. Degree of freedom is 1 for all factors.

| **Response (Abbreviation)** | **Statistic** | **Treatment** | **Climate-Origin** | **Treatment x Climate-Origin** |
| --- | --- | --- | --- | --- |
| Plant height | F | 100.119 | 0.031 | 4.566 |
| (Hmax) | P | 0.000*** | 0.861 | 0.035* |
| Total leaf area | F | 53.922 | 9.359 | 14.249 |
| (TLA) | P | 0.000*** | 0.003** | 0.000*** |
| Basal diameter | F | 101.135 | 1.936 | 1.478 |
| (BD) | P | 0.000*** | 0.167 | 0.227 |
| Wood density | F | 33.097 | 1.153 | 0.259 |
| (WD) | P | 0.000*** | 0.286 | 0.612 |
| Specific leaf area | F | 1.111 | 1.104 | 5.860 |
| (SLA) | P | 0.324 | 0.298 | 0.019* |
| Leaf dry matter content | F | 8.903 | 4.089 | 0.059 |
| (LDMC) | P | 0.018* | 0.048* | 0.809 |
| Stomatal conductance | F | 84.837 | 1.030 | 0.066 |
| (gs) | P | 0.000*** | 0.317 | 0.798 |
| Predawn water potential | F | 5.363 | 0.239 | 0.654 |
| (PD) | P | 0.028* | 0.646 | 0.425 |
| Midday water potential | F | 7.634 | 0.880 | 0.199 |
| (MD) | P | 0.009** | 0.392 | 0.658 |
| Relative Chlorophyll | F | 1.939 | 3.240 | 0.547 |
| (RC) | P | 0.212 | 0.082 | 0.466 |

Significant codes: 0 ‘***’ 0.001 ‘**’ 0.01 ‘*’ 0.05.
