# Supplementary figures and images for: Contributions of phenotypic integration, plasticity and genetic adaptation to adaptive capacity relating to drought in Banksia marginata (Proteaceae)
Source: Front Plant Sci. 2023 Apr 21;14:1150116. doi: 10.3389/fpls.2023.1150116 (PMC10160485; doi:10.3389/fpls.2023.1150116)

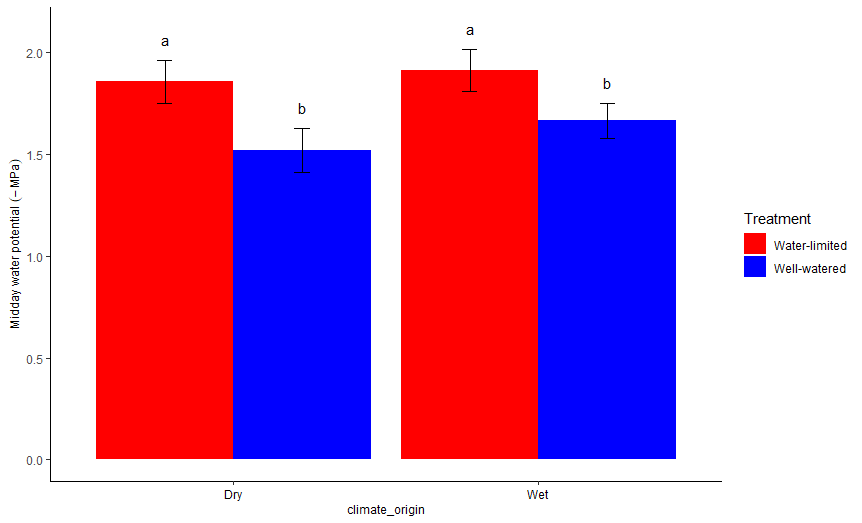

Supplement: Supplementary Figure 1 — Mid-day water potential (MD) expressions of wet and dry climate-origin populations under water-limited (WL) and well-watered (WL) treatments. [file Image_1.tiff]
